# Neurodevelopmental alcohol exposure elicits long-term changes to gene expression that alter distinct molecular pathways dependent on timing of exposure

**DOI:** 10.1186/1866-1955-5-6

**Published:** 2013-03-13

**Authors:** Morgan L Kleiber, Katarzyna Mantha, Randa L Stringer, Shiva M Singh

**Affiliations:** 1 Molecular Genetics Unit, Department of Biology, University of Western Ontario, London, Ontario, N6A 5B7, Canada

**Keywords:** Fetal alcohol spectrum disorders, Development, Brain, Mouse, Gene expression, Expression array

## Abstract

**Background:**

Maternal alcohol consumption is known to adversely affect fetal neurodevelopment. While it is known that alcohol dose and timing play a role in the cognitive and behavioral changes associated with prenatal alcohol exposure, it is unclear what developmental processes are disrupted that may lead to these phenotypes.

**Methods:**

Mice (n=6 per treatment per developmental time) were exposed to two acute doses of alcohol (5 g/kg) at neurodevelopmental times representing the human first, second, or third trimester equivalent. Mice were reared to adulthood and changes to their adult brain transcriptome were assessed using expression arrays. These were then categorized based on Gene Ontology annotations, canonical pathway associations, and relationships to interacting molecules.

**Results:**

The results suggest that ethanol disrupts biological processes that are actively occurring at the time of exposure. These include cell proliferation during trimester one, cell migration and differentiation during trimester two, and cellular communication and neurotransmission during trimester three. Further, although ethanol altered a distinct set of genes depending on developmental timing, many of these show interrelatedness and can be associated with one another via ‘hub’ molecules and pathways such as those related to *huntingtin* and *brain-derived neurotrophic factor*.

**Conclusions:**

These changes to brain gene expression represent a ‘molecular footprint’ of neurodevelopmental alcohol exposure that is long-lasting and correlates with active processes disrupted at the time of exposure. This study provides further support that there is no neurodevelopmental time when alcohol cannot adversely affect the developing brain.

## Background

Maternal ethanol consumption is known to be detrimental to the carefully-organized molecular processes that guide the neurodevelopment of a fetus. It can lead to a variety of physiological and neurological consequences, collectively known as fetal alcohol spectrum disorders (FASDs) [1-3]. Given that approximately one-half of North American women drink at least occasionally, and that half of all pregnancies are unplanned, it is likely that at least one-quarter of all infants born have been exposed to alcohol at least once during gestation [4], making FASD one of the most common and financially costly sources of developmental disability [5-7]. Further, the effects of prenatal alcohol exposure are long-lasting, drastically affecting the developmental and socioeconomic trajectory of the affected individual [8-10]. Our understanding of how neurodevelopmental alcohol exposure can cause these persistent changes in cognitive function, however, is not understood.

Most of the existing literature on FASD has focused on using animal models to understand the immediate effects of developmental ethanol exposure. These studies have shown that ethanol exposure is able to alter gene expression in the developing brain, both immediately following acute high-dose exposure [11-13] as well as in the adult brain following moderate chronic neurodevelopmental exposure [14,15] and binge exposures [16,17], altering genes involved in cell survival, cell signaling, and neurotransmission. However, these studies often use variable treatment models with regard to dosage, timing, and assessment of a given molecular or behavioral phenotype, making comparisons between studies difficult. Also, while these studies often report acute and short-term changes to the transcriptome, it is less clear what the long-term consequences of ethanol exposure are and how exposure may alter neural function across the lifetime of the exposed individual.

Previously, we have shown that voluntary maternal ethanol consumption during gestation leads to changes in brain gene expression in exposed adult mice [15]. While the results identified a number of genes that have previously been implicated in FASD-relevant neurobehavioral phenotypes such as cognitive function, anxiety, attention deficit hyperactivity disorder, and mood disorders, the effects of this moderate exposure throughout pregnancy were subtle, making the identification of statistically significant gene changes, and therefore affected pathways, challenging. Further, this voluntary maternal consumption paradigm (consisting of continuous maternal free access to a 10% ethanol solution throughout gestation) did not allow us to assess the effect of timing of ethanol exposure on the specific biological processes that were occurring at each neurodevelopmental stage. The results do, however, suggest that maternal alcohol consumption is able to leave a complex ‘footprint’ that remains long after the cessation of alcohol exposure. These changes are persistent and alter molecular pathways important for adult brain function.

Recently, we have evaluated the effects of a binge-like ethanol exposure in a C57BL/6J (B6) mouse model at three specific gestational or postnatal times roughly corresponding to the occurrence of human trimesters one, two, and three [18-20]. This treatment paradigm resulted in offspring showing many phenotypes relevant to FASD including delay of achievement of basic neuromuscular coordination and reflexes, altered activity and anxiety-related traits, and spatial learning and memory impairments [21]. Interestingly, although the treatments generally produced similar sets of behavioral changes, a closer examination of each specific phenotype revealed that their occurrence and severity were dependent on the developmental timing of the ethanol exposure. This follows recent studies that have started to evaluate the relationship between exposure at various developmental stages and the resulting phenotypic effects. Behaviorally, it has been found that early trimester-three equivalent exposure was more detrimental to adult trace conditioning than later treatments [22]. Also, a recent study by Lipinski *et al.*[23] revealed that small changes in exposure during early gestation led to differences in craniofacial morphology in a mouse model of fetal alcohol syndrome (FAS) that, interestingly, correlated with specific brain abnormalities.

This study seeks to extend our previous studies evaluating the long-term consequences of neurodevelopmental alcohol exposure. Specifically, we investigate the timing effects of a consistent dose of ethanol administered at the mouse equivalent of the first, second, or third human trimesters. Our objective is to identify the long-term effects of disrupting neurobiological processes occurring at these three times by evaluating the residual pattern of altered genes, pathways, and gene networks within the adult brain. We show that there are some common pathways that are affected by ethanol exposure, regardless of developmental timing. Also, our data suggest that ethanol exposure tends to disrupt critical molecular processes that are known to be actively occurring at each specific developmental time. These processes remain altered at least into adulthood, when the behavioral phenotypes that may be driven by these molecular alterations can affect an individual’s overall socioeconomic success [3,9,24]. It is hoped that this study provides a broad analysis of the long-term molecular effects of developmental ethanol exposure, and that it allows for a comparison of the susceptibility of particular processes at three critical neurodevelopment times to alcohol teratogenicity.

## Methods

### Animals and ethanol treatment

All animal protocols were approved by the Animal Use Subcommittee at the University of Western Ontario (London, ON, Canada) and complied with the ethical standards established by the Canadian Council on Animal Care. C57BL/6J (B6) mice were originally obtained from Jackson Laboratories (Bar Harbor, ME, USA) and subsequently maintained at the Health Sciences Animal Care Facility at the University of Western Ontario. Males and females were housed in same-sex colonies with *ad libitum* access to water and food. Cages and bedding, including access to environmental enrichment, were standardized between cages. Colonies were kept in a controlled environment on a 14/10-h light/dark cycle at a temperature of 21°C to 24°C with 40% to 60% humidity.

Female mice of approximately 8 weeks of age were time-mated overnight with 8- to 12-week old males. During gestation, dams were housed individually in standard cages. Six treatment times were selected to approximate ethanol exposure occurring at the human first, second, and third trimesters: dam treatment at embryonic days (E) 8 and 11 (human trimester one equivalent), E14 and 16 (human second trimester equivalent), and pup treatment on postnatal days (P) 4 and 7 (human third trimester equivalent) [19,25]. Each mouse (dam or pup) was treated on two treatment. To model punctuated high-blood alcohol (binge-like) exposure at these specific stages, dams (trimesters one and two) or pups (trimester three) were injected subcutaneously with 2.5 g/kg of ethanol in 0.15 M saline at 0 h and 2 h. This method has been previously reported and induces a peak blood alcohol level of over 0.3 g/dl for 4 to 5 h following injection, and is sufficient to induce neuronal apoptosis and result in FASD-related behaviors [21,26,27]. Control dams and pups were injected with saline alone, and where possible, mice were matched across treatments for weight. Pups were weaned into same-sex colonies of two to four mice at P21 to P25 and raised under standard housing conditions.

### RNA isolation and microarray hybridization

At P60, male offspring from the above treatment models were sacrificed by carbon dioxide asphyxiation and whole brain tissue (all structures including the olfactory bulb to the medulla) was isolated, snap-frozen in liquid nitrogen, then stored at -80°C until RNA isolation. Total RNA was isolated using Trizol® (Invitrogen, Carlsbad, CA, USA) according to the manufacturer’s instructions and cleaned using RNeasy Mini kit (QIAGEN, Valencia, CA, USA). The quality and quantity of RNA was assessed using the Agilent 2100 Bioanalyzer (Agilent Technologies Inc., Palo Alto, CA, USA) and a NanoDrop ND-1000 spectrophotometer (Thermo Fisher Scientific Inc., Wilmington, DE, USA). Each biological replicate consisted of equal quantities of RNA from three non-littermate males pooled to reduce litter effects. Two biological replicates per treatment group were used (*n*=12 mice per treatment time). All RNA labeling and hybridization steps were performed at the London Regional Genomics Centre (Robarts Research Institute, London, ON, Canada). Briefly, single-stranded complementary DNA (sscDNA) was synthesized using 200 ng of total RNA using the Ambion WT Expression Kit for Affymetrix GeneChip Whole Transcript WT Expression Arrays (Applied Biosystems, Carlsbad, CA, USA) and the Affymetrix GeneChip WT Terminal Labeling kit and hybridization manual (Affymetrix, Santa Clara, CA, USA). First-cycle cDNA was transcribed *in vitro* to cRNA, and used to synthesize 5.5 μg of sscDNA that was subsequently end-labeled and hybridized for 16 h at 45°C to Affymetrix Mouse Gene 1.0 ST arrays. For each treatment time, arrays (two control and two ethanol-exposed) were used for a total of 12 arrays. Liquid-handling steps were performed by a GeneChip Fluidics Station 450 and arrays were scanned using the GeneChip Scanner 3000 using Command Console v1.1 (Affymetrix, Santa Clara, CA, USA).

### Microarray data analysis

Probe level (.CEL) data were generated using Affymetrix Command Console v1.1 and probes were summarized to gene-level data using Partek Genomics Suite software v.6.6 (Partek Inc., St. Louis, MO, USA). Array data from all treatment times (12 arrays) were included in a single analysis. Data were background corrected, quantile-normalized, summarized using the GeneChip-Robust Multiarray Averaging (GC-RMA) algorithm to take into account probe GC-content [28], and log_2_-transformed. The Partek Suite was also used to determine gene-level ANOVA *P* values and fold changes. Given that prenatal ethanol exposure produces mostly subtle long-term changes in gene expression [15], genes meeting the criteria of a 1.2-fold change with a FDR-corrected *P* value <0.05 were considered for further analysis. Unannotated genes and standards were removed from the gene lists used for clustering and pathway analyses. These significant genes were subjected to hierarchical clustering analysis using Euclidean distance and average linkage to assess the consistency across replicates and to evaluate the general trends in changes to gene expression across each treatment. All data files from the array experiments have been deposited in the National Center for Biotechnology Information Gene Expression Omnibus and can be found under the accessions GSE34469 and GSE34549. Complete lists of differentially expressed genes identified for each of the three treatments are given in Additional file 1: Table S1.

### Gene ontology and gene network analysis

To evaluate genes and processes that may be involved in a general ethanol response regardless of developmental timing, a list containing genes that were differentially expressed across multiple treatments was generated. This list was analyzed using the functional annotation and clustering software tool available through the online Database for Annotation, Visualization and Integrated Discovery (DAVID) Bioinformatics Resources v.6.7 [29,30]. Gene Ontology (GO) terms and Swiss-Prot and Protein Information Resource (SP PIR) keywords were used to cluster genes. Non-redundant terms with *P* <0.05 were reported.

To identify over-represented GO terms associated with genes showing altered expression for individual trimester treatments, Ingenuity Pathway Analysis v.9 (IPA) (Ingenuity Systems, Redwood, CA, USA; http://www.ingenuity.com) was used to cluster genes based on biological function. Average fold-change and *P* values of each gene were included in the analysis. As most genes identified were downregulated, both up- and downregulated genes were assessed concurrently. Redundant GO categories (those containing the same molecules with closely related functions) were removed. Biological functions annotations with a *P* <0.05 were included. IPA was also used to generate gene networks between genes possessing annotated functional relationships with other genes, proteins, and molecules. Networks showing a cutoff significance score of 3 or more (*P* <0.001) were reported.

### Quantitative RT-PCR

RNA extracts not used for array hybridization were used to confirm the expression of selected genes by real-time RT-PCR. Complementary DNA (cDNA) was synthesized using 2 μg of total brain RNA using a High-Capacity cDNA Reverse Transcription kit (Applied Biosystems, Foster City, CA, USA) according to the manufacturer’s protocol. Gene-specific TaqMan® Assay Reagents and TaqMan Gene Expression Assay products were used on a StepOne Real Time PCR System (all Applied Biosystems, Foster City, CA, USA). Primers and FAM-labeled probes for *Tnfrsf19* (E8/11) *Cdkn1a*, *Manf*, *Htr5a* (E14/16), *Cnr1*, and *Grin2b* (P4/7) were obtained from Applied Biosystems Inventoried Assays and used as per the manufacturer’s instructions. These genes were selected on the basis of their functional relevance to cell stress and survival pathways (*Cdkn1a*, *Tnfrsf19*) or neurological function (*Manf*, *Htr5a*, *Grin2b*, *Cnr1*). Reactions were multiplexed with *Gapdh* using gene-specific primers and a VIC-labeled probe. Reactions were run following a standard ramp speed protocol using 10 μLvolumes. PCR cycling consisted of a 10 min initiation at 95°C, followed by 40 cycles consisting of a 15 s denaturation at 95°C and an anneal and extension at 60°C for 60 s. All experiments included six biological replicates per treatment and three technical replicates per sample. Relative expression was calculated according to the comparative C_T_ method [31] using StepOne™ v2.0 software (Applied Biosystems) and analyzed using SPSS v.16 (SPSS Inc., Chicago, IL, USA). All PCR data are reported as mean ± SEM relative expression values. Significant differences were assessed using a student’s *t*-test using SPSS v.16 (SPSS Inc, Chicago, IL, USA).

## Results

### Neurodevelopmental timing of ethanol exposure dictates the pattern of gene expression changes in the adult brain

**Figure 1:**
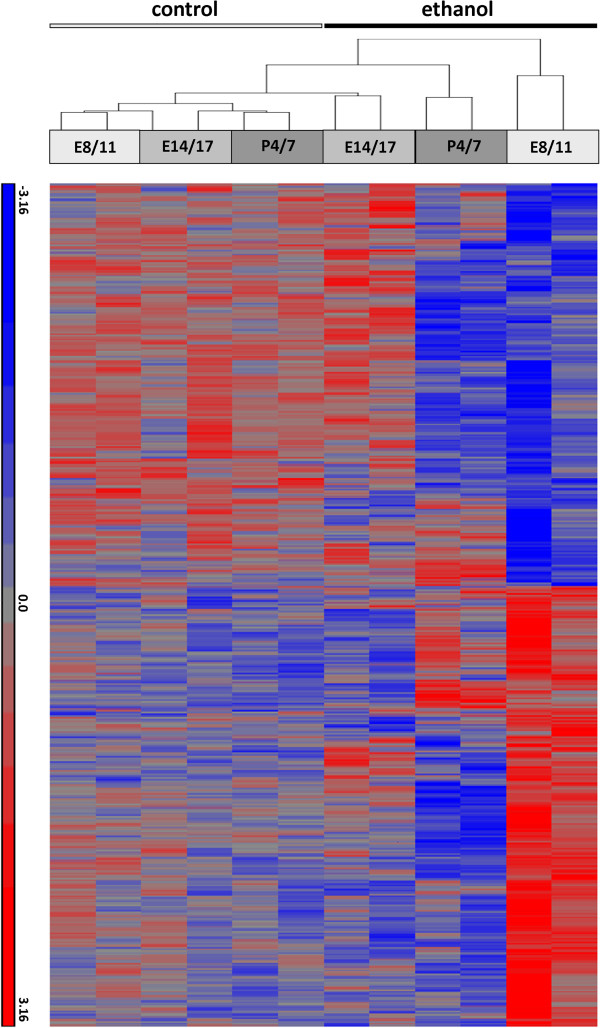
**Heat map representing hierarchical clustering of arrays based on the normalized intensity of the probe sets.** Control and ethanol-treated samples are indicated (*n*=2), with each replicate consisting of RNA pooled from three male mice obtained from six litters. Treatment times are also indicated. Heat map was generated by Partek® Genomics Suite software based on ANOVA-calculated significance levels at a fold-change cutoff of 1.2 and a FDR-corrected *P* value <0.05.

**Figure 2:**
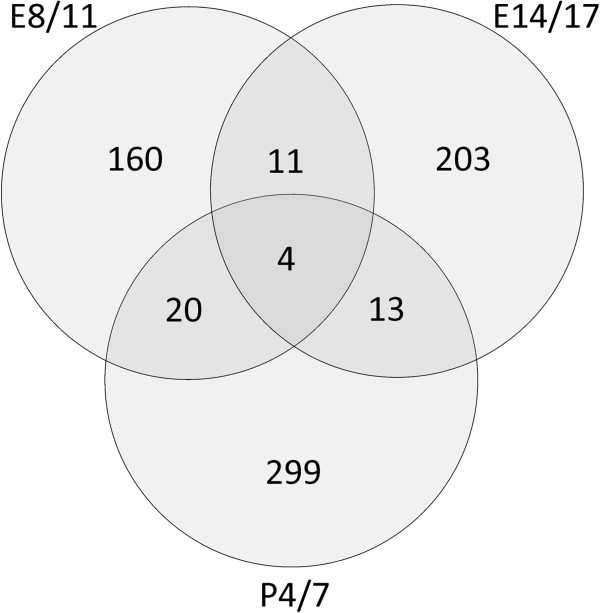
Venn diagram indicating the number of differentially expressed genes identified by each treatment paradigm (E8/11, E14/16, P4/7), including the number of genes that overlapped between multiple treatments.

We first assessed the overall patterns of gene expression in three groups of adult (P60) mice subjected to binge-like ethanol exposures at E8 and 11 (E8/11) (first human trimester equivalent), E14 and 16(E14/16) (second human trimester equivalent), or P4 and 7 (P4/7) (third human trimester equivalent) by performing a hierarchical clustering analysis of the signal ratios of all arrays. The heat map representing array clustering based on normalized probe intensity showed that the control chips cluster closely despite some inter-individual variation, as expected (Figure 1). The results also showed that the pattern of adult brain gene expression following ethanol exposure during the three developmental times was variable and dependent on the timing of the treatment, as evidenced by the distinct branches for each treatment paradigm. Neurodevelopmental ethanol exposure at E8/11, E14/16, and P4/7 resulted in the altered expression of 195 genes, 231 genes, and 336 genes, respectively (fold change >1.2, FDR-corrected *P* <0.05) in the adult brain. These changes ranged from a +3.3-fold increase from matched controls (*Proline rich protein 2* following P4/7 treatment) to -3.2-fold decrease (*Stomatin (Epb7.2)-like 3* following E14/16 treatment). Further, the distribution of up- and downregulated genes were not equal with only 61 genes (31%), 54 genes (23%), and 15 genes (4%) showing increased expression as compared to controls following ethanol treatment at E8/11, E14/16, and P4/7, respectively. There was also little overlap between genes altered across the three treatment times (Figure 2) with 82%, 88%, and 89% of the genes affected specific to a single one treatment time. Also, 48 genes were found to be altered by at least two treatments, with four (*Gmze*, *Hsf1*, *Rhog*, *Trdn*) altered by ethanol exposure by all three (Table 1, Figure 2). Full gene lists can be found in Additional file 1: Table S1.

**Table 1:** Common genes altered in the adult (P60) brain by different ethanol treatments during neurodevelopment

**Accession no.**	**Gene symbol**	**Gene name**	**Chr.**	**Fold change per treatment time**^**a**^		
				**E 8/11**	**E 14/16**	**P 4/7**
NM_019816	Aatf	Apoptosis antagonizing transcription factor	11 B5	-1.20	ns	-1.32
NM_001190371	Ankrd29	Ankyrin repeat domain 29	18 A1	-1.24	-1.2	ns
NM_145990	Cdk5rap2	CDK5 regulatory subunit associated protein 2	4 C2	-1.26	NS	-1.26
NM_198019	Cep78	Centrosomal protein 78	19 A	ns	-1.22	-1.37
NM_133840	Clp1	Cleavage and polyadenylation factor I subunit, homolog (S. cerevisiae)	2 D	ns	-1.21	-1.2
NM_027468	Cpm	Carboxypeptidase M	10 D2	-1.29	1.28	ns
NM_001163026	Dnajc13	DnaJ (Hsp40) homolog, subfamily C, member 13	9 F1	ns	1.24	-1.21
NM_008929	Dnajc3	DnaJ (Hsp40) homolog, subfamily C, member 3	14 E4	-1.21	ns	-1.38
NM_028133	Egln3	EGL nine homolog 3 (C. elegans)	12 C1	ns	-1.28	1.41
NM_153782	Fam20a	Family with sequence similarity 20, member A	11 E1	-1.24	1.21	ns
NM_010240	Ftl1	Ferritin light chain 1	7 B4	-1.26	ns	-1.51
NM_001166065	Gcnt4	Gcnt4 glucosaminyl (N-acetyl) transferase 4, core 2 (beta-1,6-N-acetylglucosaminyltransferase)	13 D1	ns	-1.24	-1.44
NM_011937	Gnpda1	Glucosamine-6-phosphate deaminase 1	18 B3	-1.21	ns	-1.48
NM_001013385	Grm4	Glutamate receptor, metabotropic 4	17 A3	-1.33	ns	-1.38
AK138760	Gtf3c2	General transcription factor IIIC, polypeptide 2, beta	5 B1	-1.33	1.37	ns
NM_010373	Gzme	Granzyme E	14 C3	1.28	-1.21	-1.21
NM_173400	Haus6	HAUS augmin-like complex, subunit 6	4 C4	1.2	ns	-1.42
NM_175256	Heg1	HEG homolog 1 (zebrafish)	16 B3	-1.2	ns	-1.23
NM_178200	Hist1h2bm	Histone cluster 1, H2bm	13 A2-3	ns	1.27	-1.53
NM_175653	Hist1h3c	Histone cluster 1, H3c	13 A2-3	-1.31	ns	-1.3
NM_001024720	Hmcn1	Hemicentin1	1 G1	1.2	ns	-1.28
NM_008296	Hsf1	Heat shock factor 1	15 D3	-1.25	1.22	-1.22
NM_054079	Iltifb	Interleukin 10-related T cell-derived inducible factor beta	10 D2	-1.22	ns	-2.2
NM_201531	Kcnf1	Potassium voltage-gated channel, subfamily F, member 1	12 A1.1	-1.31	-1.21	ns
NM_172871	Klhl9	Kelch-like 9 (Drosophila)	4 C4	ns	-1.21	-1.31
NM_011945	Map3k1	Mitogen-activated protein kinase kinase kinase 1	13 D2	ns	-1.2	-1.5
BC132343	Mars2	Methionine-tRNA synthetase 2 (mitochondrial)	1 C1	ns	-1.22	-1.62
ENSMUST00000001455	Mef2d	Myocyte enhancer factor 2D	3 F1	-1.21	ns	-1.26
NR_029580	Mir194-1	microRNA 194-1	1	1.22	ns	1.5
NM_028901	Myo18b	Myosin XVIIIb	5 F	1.26	-1.26	ns
NM_153578	Nipa1	Non-imprinted in Prader-Willi/Angelman syndrome 1 homolog (human)	7 C	-1.28	ns	-1.22
NM_001164035	Ntf3	Neurotrophin 3	6 F3	-1.41	ns	-1.58
NM_178668	Pde12	Phosphodiesterase 12	14 A3	ns	-1.22	1.25
NM_009402	Pglyrp1	Peptidoglycan recognition protein 1	7 A3	−1.31	ns	−1.31
NM_001081307	Ppp1r12b	Protein phosphatase 1, regulatory (inhibitor) subunit 12B	1 F	ns	-1.21	-1.29
NM_031499	Prp2	Proline rich protein 2	6 G1	1.68	ns	3.3
NM_029614	Prss23	Protease, serine, 23	7 E1	1.22	-1.21	ns
NM_019566	Rhog	Ras homolog gene family, member G	7 F1	-1.22	1.21	-1.48
NM_146118	Slc25a25	Solute carrier family 25 (mitochondrial carrier, phosphate carrier), member 25	2 B	-1.21	ns	-1.35
AF357427	Snord115	Small nucleolar RNA, C/D Box 115 cluster	7	ns	1.23	1.71
NR_028275	Snord14e	Small nucleolar RNA, C/D box 14E	9	1.26	-1.27	ns
NM_025291	Sra1	Steroid receptor RNA activator 1	18 B2	-1.27	-1.22	ns
NM_033622	Tnfsf13b	Tumor necrosis factor (ligand) superfamily, member 13b	8 A1	1.21	ns	-1.23
NM_029726	Trdn	Triadin	10 A4	1.27	-1.61	-2.21
BC025894	Ubc	Ubiquitin C	5 G1	ns	1.29	-1.34
NM_016982	Vpreb1	Pre-B lymphocyte gene 1	16 A3	1.46	-1.28	ns
NM_172643	Zbtb41	Zinc finger and BTB domain containing 41 homolog	1 F	-1.22	ns	-1.32
NM_028543	Zfp763	Zinc finger protein 763	17 B1	-1.29	-1.26	ns

^a^E, embryonic day; P, postnatal day; ns, not significantly different.

### Real-time PCR validation of array results

**Figure 3:**
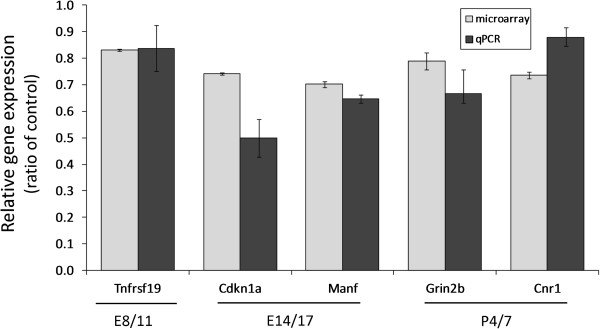
**Quantitative PCR confirmation of genes identified as altered by neurodevelopmental ethanol exposure by microarray analysis.** Expression values are shown as a ratio of the control value ± SEM. Microarray values represent the results of ANOVA comparison of two biological replicates, each consisting of the RNA from three non-littermate male mice. Quantitative PCR values were generated using six biological replicates consisting of the RNA from six ethanol-exposed males mice. Significance differences from control values were determined using unpaired *t*-tests. All bars are significantly different from their respective controls (*P* <0.05). We were unable to confirm the expression of *Htr5a* (*P* >0.05) and therefore this gene is not included in this figure.

We chose to confirm the changes in expression of five genes altered by ethanol exposure identified by the array experiments using real-time PCR to validate our array results. The real-time results and microarray fold-changes for the five genes are shown in Figure 3. For E8/11, the downregulation of tumor necrosis factor receptor superfamily, member 19 (*Tnfrsf19*) was confirmed (*P* = 0.024). We were also able to confirm the downregulation of cyclin-dependent kinase inhibitor 1A *(P21)* (*Cdkn1a*) (*P* = 0.019) and mesencephalic astrocyte-derived neurotrophic factor (*Manf*) (*P* = 0.024), both shown to be differentially expressed following ethanol treatment at E14/16. We were not able to confirm the downregulation of *Htr5a* by quantitative PCR (*P* >0.05). We also evaluated the expression of cannabinoid receptor 1 (brain) (*Cnr1*) and glutamate receptor, ionotropic, NMDA2B (epsilon 2) (*Grin2b*), both downregulated following ethanol treatment at P4/7. Real-time results confirmed the array results with both *Cnr1* (*P* = 0.0042) and *Grin2b* (*P* = 0.014) genes showing statistically significant downregulation.

### Functional annotation analysis of genes affected by ethanol treatment

Functional clustering analysis was performed using the 48 genes altered by more than one treatment paradigm (Table 1) to assess potentially common processes affected by neurodevelopmental alcohol exposure, regardless of timing. Genes were evaluated together in a single analysis using the Database for Annotation, Visualization and Integrated Discovery (DAVID ) v6.7 [29,30], which returned three significant functional annotation clusters. Most significant was the GO biological process (BP) term ‘cell death’ (*P* = 0.0047), which included the genes *Egln3*, *Gzme*, *Map3k1*, *Mef2d*, *Pglyrp1*, and *Sra1*. Also significant were genes associated with the SP PIR keyword ‘methylation’ (*P* = 0.011), consisting of *Hist1h2bm*, *Hist1h3c*, *Ubc*, and *Rhog*, and the GO BP term ‘positive regulation of gene expression’ (*P* = 0.021), consisting of *Iltifb*, *Ntf3*, *Rhog*, *Sra1*, and *Tnfsf13b*.

### Gene enrichment analysis identifies that alterations to genetic processes caused by neurodevelopmental ethanol are exposure-stage dependent

The gene lists generated from each individual treatment model were subjected to GO analysis to evaluate over-represented molecular functions and biological processes using the ‘core analysis’ function within the IPA software v.9 (Ingenuity Systems, Redwood, CA, USA). Results of this analysis indicating the most significant non-redundant GO categories and ‘functions annotations’ are shown in Table 2. The GO terms significantly associated with genes disrupted by ethanol treatment at E8/11 suggested a disruption in processes associated with cellular organization, cellular development, and cell death. The functions annotations associated with these GO categories indicate that mice exposed to ethanol at E8/11 may have deficits related to quantity of neurons and abnormal axon development, potentially leading to altered synaptic function. Interestingly, most of these GO gene enrichment clusters were driven by the presence of neurotrophin 3 (*Ntf3*).

**Table 2:** Significant gene ontology (GO) categories and molecular functions annotations for genes differentially expressed in the brain of adult (P60) mice neurodevelopmentally exposed to ethanol

**Timing of treatment**^**a**^	**GO category**	**Functions annotation**	***P *value**	**Molecules**
E 8/11	Cellular assembly and organization	Extension of axons	0.0082	Dclk1, Ntf3, Tnfrsf12a
	Protein synthesis	Translation of protein	0.0025	Eif2s1, Hspa5
	Cell-to-cell signaling and interaction	Synaptic transmission	0.0136	Egr3, Ntf3
	Tissue development	Accumulation of cells	0.0153	Cdkn1a, Ntf3
	Cell death	Cell death of sensory neurons	0.0171	Hsf1, Ntf3
	Tissue morphology	Quantity of interneurons	0.0250	Dclk1, Ntf3
E 14/16	Cellular movement	Migration of interneurons	0.00013	Dcx, Dlx1, Dlx2
	Tissue development	Development of olfactory bulb	0.0168	Dlx2, Nr2e1
	Cellular development	Differentiation of neuronal progenitor cells	0.0214	Dlx1, Dlx2
	Cell death	Cell death of sensory neurons	0.0238	Bok, Hsf1
	Neurological disease	Neurodegenerative disorder	0.0458	Cav1, Cox1, Dcx, Htr6, Pcna
	Cell morphology	Shape change of neurons	0.0473	Dcx, Hcrt
P 4/7	Cell-to-cell signaling and interaction	Synaptic transmission	0.00014	Apoe, Chrm1, Cnr1, Ephb2, Grik1, Grin2b, Htr1a, Nrgn1, Ntf3
	Lipid metabolism	Secretion of steroid	0.0018	Apoe, Pomc
	Cell-to-cell signaling and interaction	Synaptic depression	0.0133	Chrm1, Cnr1, Ephb1, Grin2b, Htt
	Cell-to-cell signaling and interaction	Plasticity of synapse	0.0144	Apoe, Grik1, Grin2b, Htt, Inppl1, Syngap1
	Cell morphology	Formation of cellular protrusions	0.0329	Apoe, Dst, Efna5, Efnb2, Egfr, Ephb1, Grm4, Htt, Kif23, Nbl1, Ntf3, Ntng2, Pcdh15, Rhog, Syne1, Syngap1, Wnt3a
	Cellular development	Proliferation of neuronal cells	0.0366	Cnr1, Irx5, Mycn, Nbn, Ntf3, Wnt3a
	Neurological disease	Gliosis of hippocampus	0.0400	Apoe, Htt

^a^E, embryonic day; P, postnatal day.

The significant GO categories identified in adult mice treated with ethanol at E14/16 involved changes to molecules involved in cellular movement, cell death, and cell morphology (Table 2). In contrast to the E8/11 treatment results, which suggested changes to processes involved in cellular survival and proliferation, the functions annotations associated with these GO categories suggested that these mice may show alterations related to neuronal migration and differentiation. These categories appeared to be driven by the altered expression of doublecortin (*Dcx*) and distal-less homeobox 1 and 2 (*Dlx1*, *Dlx2*). Interestingly, similarly to the cell death annotation in the E8/11 exposure, the ‘cell death’ function identified in the E14/16 paradigm was also driven by heat shock factor 1 (*Hsf1*).

The GO categories associated with genes altered by exposure at P4/7 were primarily associated with cell-cell signaling and communication, with alterations to genes whose functions included roles in synaptic formation, transmission, depression, and plasticity. Further, alterations to a number of molecules involved in steroid-mediated cell communication (such as glucocorticoids) were identified, as were changes in genes involved in the gliosis of the hippocampus. These molecular functions suggest that P4/7 ethanol exposure may significantly alter the synaptic function and structure of the adult brain. The molecules that show altered expression following P4/7 exposure support this, with changes to a number of critical neurotransmitter-related systems such as glutamate (*Grik1*, *Grin2b*, *Grm4*), serotonin (*Htr1a*), cannabinoids (*Cnr1*) and ephrins (*Ephb1*, *Ephb2*). Also notable is the altered regulation of apolipoprotein E (*Apoe*) and propriomelanocortin (*Pomc*) given their roles in the development and function of the hypothalamic-pituitary-adrenal (HPA) axis.

### Canonical pathways affected by neurodevelopmental ethanol exposure

Using Ingenuity® software, we also evaluated the canonical pathways that may be altered as a result of each ethanol treatment. E8/11 exposure was associated with the endoplasmic reticulum stress pathway, xenobiotic metabolism signaling, and, interestingly, glucocorticoid receptor signaling (Table 3). Also significant were phospholipase C signaling, antiproliferative role of somatostatin receptor 2, and calcium signaling. The identification of these pathways as significantly altered support that E8/11 exposure may disrupt general systems that have a pleiotropic developmental function. Canonical pathways associated with E14/16 exposure were fatty acid biosynthesis, serotonin receptor signaling, and regulation of actin-based motility by Rho (Table 3). These results are consistent with the gene ontology results and support that ethanol exposure during E14/16 may result in the long-term disruption of genes associated with brain cell proliferation and migration processes, as well as some cell-communication functions. Finally, P4/7 exposure was most significantly associated with alterations in molecules involved in glutamate receptor signaling (Table 3), also supporting the P4/7 GO results. Other developmentally-associated Canonical Pathways identified were retinoic acid-mediated apoptosis signaling, ephrin receptor signaling, and, interestingly, Circadian rhythm signaling, which was driven by the alterations to a number of glutamate receptor subunits (*Grin2b*, *Grin3b*, *Grin2c*, and *Grin2d*). P4/7 ethanol exposure also was associated with changes to the one carbon pool by folate and mTOR signaling pathways.

**Table 3:** Ingenuity® canonical pathways significantly associated with genes altered in the adult (P60) brain by neurodevelopmental alcohol exposure

**Timing of treatment**^**a**^	**Ingenuity® canonical pathway**	***P *value**	**Molecules**
E 8/11	Endoplasmic reticulum stress pathway	0.00003	Xbp1, Dnajc3, Eif2s1, Hspa5
	Xenobiotic metabolism signaling	0.00282	Sra1, Ftl, Nras, Map3k13, Camk1g, Cyp2c19, Ncor2, Mapk11
	Glucocorticoid receptor signaling	0.00501	Sra1, Nras, Hpsa1a, Taf5l, Cdkn1a, Ncor2, Hspa5, Mapk11
	Phospholipase C signaling	0.00741	Rhog, Nras, Mef2d, Myl4, Gng13, Rapgef3, Itga4
	Antiproliferative role of somatostatin receptor 2	0.0263	Nras, Cdkn1a, Gng13
	Calcium signaling	0.0269	Calr, Mef2d, Trdn, Camk1g, Myl4
E 14/16	Fatty acid biosynthesis	0.00363	Oxsm, Mcat
	Serotonin receptor signaling	0.00603	Htr5a, Htr3a, Htr6
	Regulation of actin-based motility by Rho	0.0145	Myl9, Rhog, Rnd3, Ppp1r12b
P 4/7	Glutamate receptor signaling	0.000007	Grin2b, Grin3b, Gnb3, Grid1, Grin2c, Grin2d, Grik3, Grm4, Grik1
	Retinoic acid-mediated apoptosis signaling	0.00055	Fadd, Casp3, Rarb, Tnfsf10, Rxrb, Irf1
	Ephrin receptor signaling	0.00224	Frin2b, Efnb2, Ephb1, Grin3b, Gnb3, Itsn1, Grin2c, Efna5, Grin2d, Vegfc, Figf
	Circadian rhythm signaling	0.00692	Grin2b, Grin 3b, Grin2c, ,Grin2d
	One carbon pool by folate	0.00794	Tyms, Mthfs, Mtr
	mTOR signaling	0.0288	Fau, Rhog, Rps10, Rptor, Vegfc, Figf, Insr, Rpsa

^a^E, embryonic day; P, postnatal day.

### Network analysis shows common interacting molecules regardless of developmental timing of ethanol exposure

Using the network analysis tool within the Ingenuity® software platform, we predicted interacting molecular networks to further evaluate the broader potential effects of neurodevelopmental ethanol exposure on the function of adult neurological systems. These analyses suggested that related regulatory and/or effector pathways are associated with ethanol exposure regardless of timing, as well as highlighted some differences that may be associated with timing-specific physiological or behavioral FASD-associated abnormalities.

**Figure 4:**
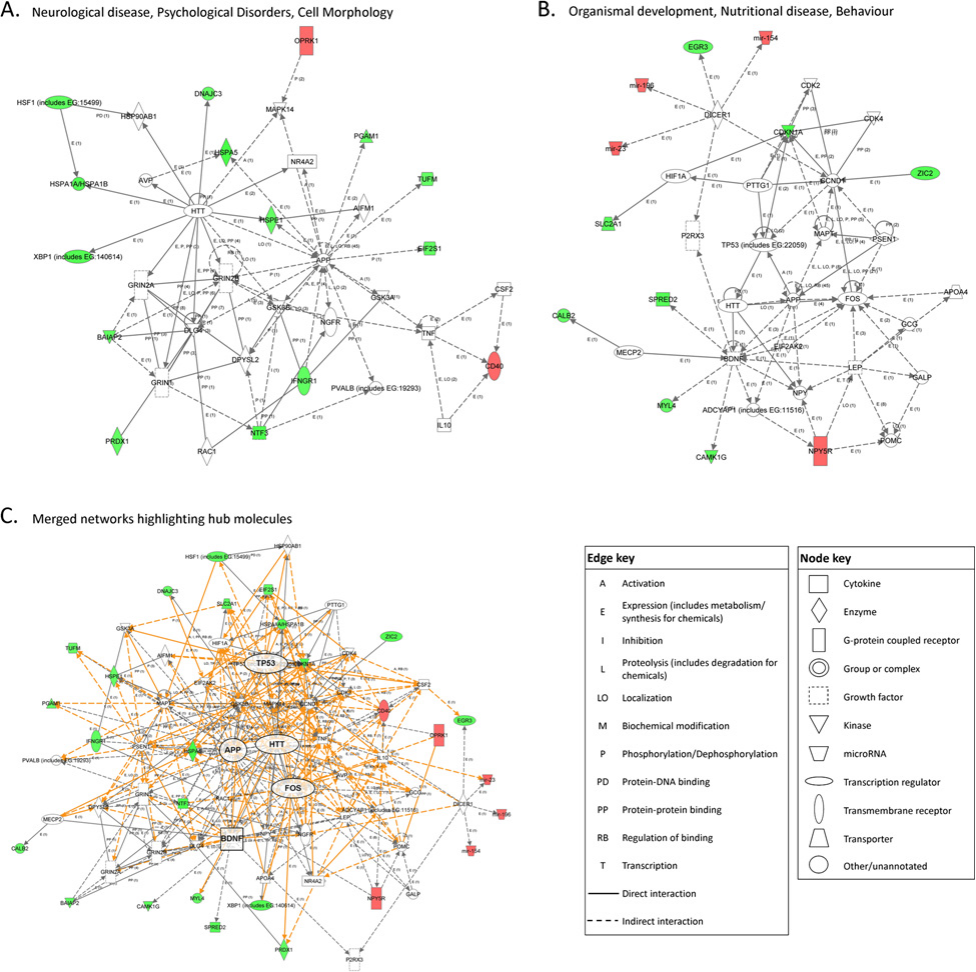
**Ingenuity pathway analysis (IPA) network analysis indicating annotated interactions between genes affected in the adult brain by ethanol exposure at E8 and 11.** Up- (red) and down- (green) regulated genes are indicated. Significant networks identified, as well as their IPA functional category, are shown in (**A**) and (**B**). (**C**) Merged image of networks shown in (A) and (B) showing the interrelatedness of the genes involved. Centralized ‘hub’ molecules linking multiple interacting genes are enlarged.

Analysis of the molecules altered following E8/11 exposure revealed two significant interacting gene networks related to ‘Neurological Disease, Psychological Disorders, and Cell Morphology’ (NPC) with 18 focus molecules, and ‘Organismal Development, Nutritional Disease, and Behavior’ (ONB) with 13 focus molecules extracted from the differentially expressed genes (Figures 4A and B). The NPC network identified a number of molecules related to glutamate signaling although the receptor subunit genes themselves were not differentially expressed. The ONB network indicated the significant interaction of altered genes with the immediate early transcription factor, FBJ murine osteosarcoma viral oncogene homolog (*Fos*), and, interestingly, three upregulated microRNAs (miRNAs). Given that these two networks contained a number of overlapping genes, the networks were merged to produce a broader view of the molecules that linked the genes altered by E8/11 ethanol exposure (Figure 4C). The merged networks revealed several ‘hub’ molecules including amyloid beta (A4) precursor protein (*App*), brain-derived neurotrophic factor (*Bdnf)*, *Fos*, huntingtin (*Htt*), and tumor protein p53 (*Tp53*).

**Figure 5:**
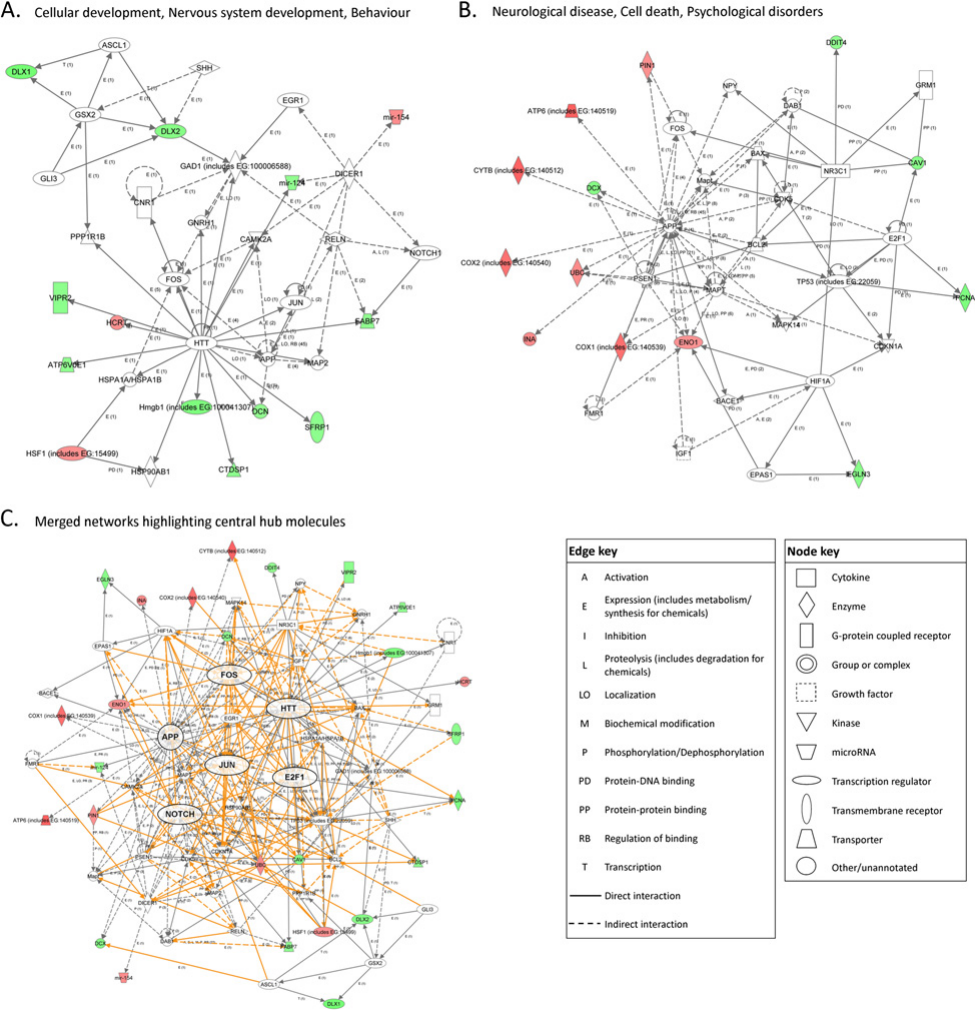
**Ingenuity pathway analysis (IPA) network analysis indicating annotated interactions between genes affected in the adult brain by ethanol exposure at E14 and 16.** Up- (red) and down- (green) regulated genes are indicated. Significant networks identified, as well as their IPA functional category, are shown in (**A**) and (**B**). (**C**) Merged image of networks shown in (A) and (B) showing the interrelatedness of the genes involved. Centralized ‘hub’ molecules linking multiple interacting genes are enlarged.

Network analysis of genes altered by E14/16 exposure revealed similarities to the E8/11 exposure results, suggesting some common effector molecules may act in response to ethanol exposure regardless of developmental timing. The most significant network functions were ‘Cellular Development, Nervous System Development and Function, Behavior’ (CNB), with 14 focus molecules, and ‘Neurological Disease, Cell Death, and Psychological Disorders’ (NCP), also with 14 focus molecules (Figures 5A and B). Again, *Htt* and *Fos* appeared as hub molecules within the CNB network, as well as Jun proto-oncogene (*Jun*), which, with *Fos*, forms the early response transcription factor Activator protein 1 (Ap-1). *App* once again formed a main hub within the NCP network, and *Fos* and *Tp53* appeared within this network similarly to the networks generated from the E8/11 data. Merging of these two networks revealed *App*, *Fos*, and *Htt* as major hubs once again (Figure 5C). Other major hubs unique to the E14/16 treatment paradigm included notch signaling, *Jun*, and the cell-cycle transcription factor *E2f1*.

**Figure 6:**
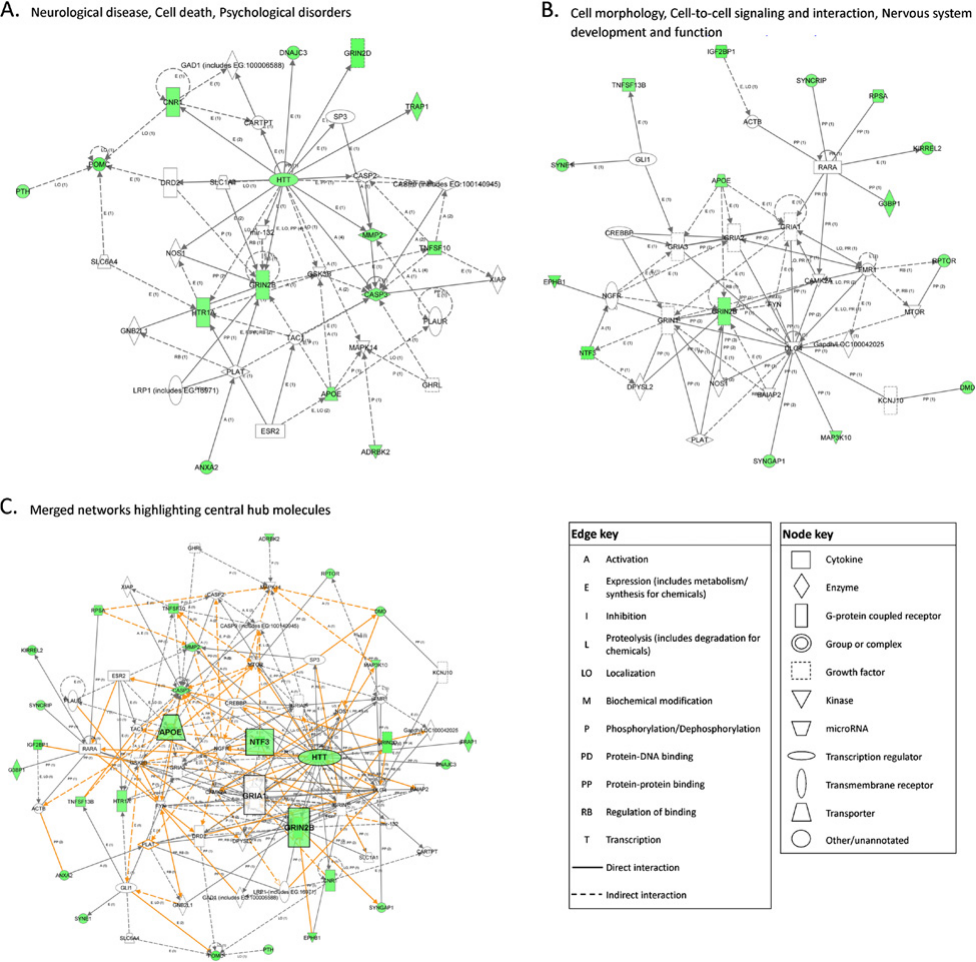
**Ingenuity pathway analysis (IPA) network analysis indicating annotated interactions between genes affected in the adult brain by ethanol exposure at P4 and P7.** Up- (red) and down- (green) regulated genes are indicated. Significant networks identified, as well as their IPA functional category, are shown in (**A**) and (**B**). (**C**) Merged image of networks shown in (A) and (B) showing the interrelatedness of the genes involved. Centralized ‘hub’ molecules linking multiple interacting genes are enlarged.

Network analysis of P4/7 ethanol exposure resulted in gene networks that included some molecules common to the E8/11 and E14/16 analyses, many of which showed altered expression in this treatment model. The two most significant associated network functions were ‘Neurological disease, Cell Death, Psychological Disorders’ (NCP) with 17 focus molecules and ‘Cell Morphology, Cell-To-Cell Signaling and Interaction, Nervous System Development and Function’ (CCN) with 14 focus molecules (Figures 6A and B). These network functions support the results of the gene ontology and pathway analysis, suggesting that P4/7 exposure may result in altered cell-cell communication and synaptic function, as well as alterations to intrinsic apoptosis cascade proteins. The NCP network contained *Htt* as a hub molecule, similar to both E8/11 and E14/16 treatment networks, which was identified as downregulated in the adult brain following P4/7 ethanol treatment. Other hub molecules included glutamate receptor, ionotropic, N-methyl D-aspartate 2B (*Grin2b*) and the apoptosis enzyme Caspase 3 (*Casp3*), both of which were also downregulated. The CCN network also contained a number of glutamate receptor subunits with *Grin2b* acting as a network hub molecule connecting multiple interacting genes. Other hub molecules included the synaptic protein discs, large homolog 4 (Drosophila) (*Dlg4)* and retinoic acid receptor, alpha (*Rara*). The merged networks again suggest *Htt* as a central hub connecting multiple molecules (Figure 6C), as well as the glutamate receptor proteins *Grin2b* and glutamate receptor, ionotropic, AMPA1 (alpha 1) (*Gria1*). Neurotrophin 3 (*Ntf3*), identified as differentially expressed in the adult brain following ethanol treatment at E8/11, was also downregulated by ethanol treatment at P4/7 and appeared as a hub within the merged gene networks, as did apolipoprotein E (*Apoe*).

## Discussion

The development of the brain is highly orchestrated, coordinated by proper gene expression in response to developmental cues and in response to the environment. It is also a remarkably vulnerable target for a variety of drugs of abuse, particularly ethanol [32,33]. This vulnerability leads to changes in brain structure and function, resulting in the long-term behavioral and cognitive changes that characterize FASD [1,3,34-36]. The manifestation and severity of specific FASD phenotypes, however, varies widely across individuals prenatally exposed to ethanol for reasons that are not entirely clear. Further, while it is known that developmental ethanol exposure leads to changes in neural molecular architecture, including changes to genome-wide gene expression [11,12,15]), how these alterations are initiated, maintained over time, and relate to specific FASD-relevant neurological phenotypes remains unknown. The results included in this report focus on the pattern of long-term changes in brain gene expression in adult B6 mice following ethanol exposure at three neurodevelopmental times corresponding to the approximate human equivalent of trimesters one, two, and three.

In a previous experiment, we have determined that the exposure paradigms used in this study (binge-like treatments at developmental days representing early, mid, and late human gestational neurodevelopmental exposure) result in behavioral phenotypes that are relevant to the abnormalities observed in individuals with FASD [21]. This study seeks to extend these findings by evaluating the genes and their associated pathways that show long-term disruption due to prenatal alcohol exposure. We do acknowledge that the neurodevelopmental timelines in rodents and humans are similar but not identical, particularly the period of synaptogenesis that occurs during the third trimester and extends postnatally in humans but occurs primarily postnatally in mice [19,37]. Also, due to these differences, two of the treatment models (ethanol exposures at E8/11 and E14/16) represent maternal treatments whereas mouse pups were administered ethanol in the trimester three model (exposure at P4/7). This may result in differences in total blood alcohol levels achieved, which is a caveat of these methods, although there is evidence that ethanol rapidly crosses across the placental barrier and can appear within the embryo within 5 minutes after maternal treatment [38]. Regardless of these potential differences in blood alcohol levels achieved, the results obtained using this mouse model may be an effective means to parse the long-term consequences of fetal alcohol exposure to the developing brain [18,39].

### Ethanol-induced long-term changes to gene expression are subtle, multifactorial, and timing-dependent

The clustering of gene expression signal intensities of the arrays representing the control and ethanol-treated samples (Figure 1) suggested that alcohol exposure during neurodevelopment significantly altered the transcriptome of the adult brain, regardless of timing. Maternal care differences were not assessed in this study and although we and others have previously observed that minor, if any, alterations to maternal care occur following gestational alcohol exposure [40-42], these finding are predominantly based on voluntary maternal consumption paradigms and not binge-like exposures. It is possible, then, that maternal care differences between ethanol-treated and control dams may contribute to the observed gene expression differences. However, there was very little overlap between the genes identified by each of the ethanol-treatment times (Table 1, Figure 2), suggesting that a major contributor to the set of genes affected was the timing of exposure. This may be attributed to the considerable changes that occur to the brain transcriptome across development, resulting in a different repertoire of processes on which ethanol may act and leading to a very different footprint of genes that remain altered into adulthood.

### Genes that remain altered by neurodevelopmental alcohol exposure may reflect predominant biological processes occurring at the time of exposure

The results included in Table 1 and Figure 2 show that only a small number of genes were altered by ethanol by multiple treatment models. In general, these genes represent pleiotropically-acting molecules associated with cell death, regulation of gene expression, and, interestingly, methylation. Such results may argue that the most common and timing-independent response to alcohol is cellular stress, potentially leading to apoptosis at the time of exposure. The long-term changes to these genes may suggest that the surviving cells retain a ‘memory’ of exposure and may show altered response to subsequent exposures. This is supported by evidence that prenatal ethanol exposure can result in heightened ‘sensitization’ of adult neurons to ethanol [43,44], which may be a contributing factor to fetal alcohol exposure and increased risk for alcohol abuse [45]. These genes, however, represent only a small proportion of the genes detected, which suggests that the effect of developmental alcohol exposure may be dictated by the interaction between ethanol and the specific biological processes occurring at the time of exposure.

### Ethanol exposure during trimester one alters genes associated with tissue morphology

We performed GO analysis on the gene lists generated by each treatment model (E8/11, E14/16, and P4/7) in order to identify the overrepresented biological functions altered in each paradigm. Interestingly, genes affected in each of the three treatments showed relevance to predominant biological processes occurring at the developmental stage of exposure. For example, E8/11 exposure was associated with alterations to genes involved in cellular assembly, proliferation, differentiation, cell death, and tissue morphology (Table 2), even in the adult brain. Developmentally, ethanol-induced disruptions in decisions regarding the ‘accumulation of cells’ and ‘quantity of interneurons’ during early gestation could lead to alterations in the number of cells that exist in certain brain regions, perhaps through inhibition of mitosis or by promoting inappropriate apoptosis, but it is unclear what effect the alteration of these genes may have in the adult brain. The significant functions annotations for trimester one exposure appear to be driven primarily by the altered expression of Neurotrophin 3 (*Ntf3*). Disruptions in *Ntf3* expression during development are detrimental given its canonical role as a neuronal survival factor [46,47] and regulator of axonal growth and guidance, synaptic structure, and synaptic plasticity [48-51]. In addition to having developmental roles, reduced *Ntf3* expression in the adult brain has been shown to be associated with deficits in spatial learning, long-term potentiation impairments, and increased anxiety-related traits [52-54]. Canonical pathways analysis indicated alterations to genes involved in endoplasmic reticulum (ER) stress response and such as the downregulation of DnaJ (Hsp40) homolog, subfamily C, member 3 (*Dnajc3*), also known as *p58IPK.* Cells showing decreased expression of this molecular chaperone protein are more prone to ER stress-induced apoptosis [55,56]. These results suggest that, beyond inducing cell death upon immediate exposure, early gestational ethanol treatment may lead to increased cellular vulnerability to other insults in surviving adult neurons.

### Ethanol exposure during trimester two affects genes involved in cellular differentiation and migration

In contrast to E8/11 exposure, GO analysis of genes affected by ethanol treatment at E14/16 revealed the alteration of genes developmentally involved with cell migration, differentiation, and morphology (Table 2), processes that occur rapidly during this developmental stage in many brain regions [20,39,57]. Physiologically, ethanol exposure during trimester two has been associated with abnormal cell division and increased the appearance of radial-glia-like precursors which dictate neural stem cell migration from the ventricular zone (VZ) to the subventricular zone (SVZ) during early differentiation [58,59]. This leads to the depletion of certain susceptible cell types in the VZ and a corresponding abnormal increase of cells in the SVZ and may lead to the cortical heterotopias associated with FASD. Interestingly, the homeobox transcription factors distal-less homeobox 1 and 2 (*Dlx1* and *Dlx2*) as well as doublecortin (*Dcx*) were identified as altered in the adult brain following E14/16 exposure. *Dcx* has been previously implicated in alcohol’s effects on both the developing and the adult brain [60-62], while *Dlx1* and *Dlx2* are the earliest *Dlx* genes to be expressed in the SVZ and are critical regulators of interneuron differentiation and migration in the developing telencephalon [63-65]. They are also responsible for the majority of GABAergic neurons in the mammalian neocortex and are therefore crucial for the regulation of the development of inhibitory neocortical circuitry [66,67]. IPA canonical pathway analysis also associated E14/16 exposure with changes to genes involved in serotonin signaling (Table 3). Indeed, three serotonin receptor subunits were identified as altered: 5-hydroxytryptamine (serotonin) receptor 5A (*Htr5a*), *Htr3a*, and *Htr6*. Reduced density of serotonin neurons have been previously associated with prenatal alcohol exposure [68-70]. Further, low serotonin signaling, including that caused by neurodevelopmental alcohol exposure, has been implicated in a number of phenotypic outcomes relevant to FASD such as increased impulsivity, aggression, hopelessness, exploratory activity, and risk for stress and anxiety-related traits due to altered hypothalamic-pituitary-adrenal axis function [71-75].

### Early postnatal ethanol exposure altered genes associated with synaptic function in the adult brain

Spanning approximately the first 2 weeks of postnatal mouse development, the ‘brain growth spurt’ is a period of extensive synaptogenesis and growth within the cortex, hippocampus, and corpus callosum [18,20]. This stage of development, equivalent to approximately the third trimester of human neurodevelopment [18,19], determines much of the extent of the brain’s neural circuitry, including the maintenance or pruning of synaptic connectivity in multiple brain regions. Disruptions to these processes, such as by ethanol, may result in inappropriate synapse formation and interfere with synaptic transmission, potentiation, and plasticity in adulthood [76-79]. GO analysis of P4/7 ethanol exposure suggested that many genes altered in the adult brain by this treatment model were involved in biological processes relevant to synaptic function. A number of glutamate receptor subunits were identified, known to be critical in the formation and maintenance of synapses, synaptic plasticity, and long-term potentiation (LTP) [80-85]. Also of note is the downregulation of Eph receptor B1 and B2 (*Ephb1* and *Ephb2*), known to have classic functions in axon guidance and brain region boundary formation [86] as well as potentially controlling many other aspects of excitatory synaptic transmission and plasticity [87-89]. Interestingly, ethanol exposure at P4/7 also resulted in altered steroid secretion due to the downregulation of both apolipoprotein E (*Apoe*) and proopiomelanocortin (*Pomc*), molecules that are essential for proper hypothalamic-pituitary-adrenal (HPA) axis function. *Apoe* deficiency is associated with age-related neurodegeneration and cognitive decline [90,91]. Phenotypically, *Apoe* null mice show increased anxiety in the elevated plus maze but reduced activity in a novel open field environment, which coincides with increased plasma corticosterone levels and impaired performance in spatial learning tests [90,92]. Contrastingly, *Pomc* has been shown to be essential for postnatal adrenal maturation, with *Pomc* null mice showing no production of corticosterone [93]. Given this, it is certainly possible that long-term alterations in the expression of these genes may contribute to phenotypes such as cognitive abnormalities and increased stress reactivity observed individuals with FASD. The IPA canonical pathway analysis reflected the results of the GO analysis, with genes associated with glutamate receptor signaling and ephrin receptor significantly represented. Genes associated with retinoic acid-mediated apoptosis signaling were also altered, a finding which is supported by other studies that report that prenatal ethanol exposure can lower retinoic ethanol receptor function and elevate retinoic acid levels beyond normal physiological levels [94-96]. Folate metabolism has also been implicated in FASD phenotypes as folic acid supplementation has been shown to ameliorate some of the effects of prenatal alcohol consumption [97,98]. Finally, although it is known that the hypothalamus, including the control of circadian ‘clock genes’, is disrupted by neurodevelopmental alcohol exposure [14,99,100], the identification of circadian rhythm signaling is interesting given that disturbed sleep patterns is a reported but relatively unexplored consequence of fetal alcohol exposure. This may be due to the disruption of corticotrophin-releasing hormone neurotransmission due to abnormal synaptic tract formation, but this potential consequence associated with trimester three ethanol exposure requires further investigation.

### Common molecular pathways may mediate the effect of ethanol on neurodevelopment despite execution through trimester-specific gene sets

As a final part of this study, we conducted IPA network analysis initially to evaluate further differences to adult brain gene expression following ethanol treatment at various neurodevelopmental stages. Evaluation of these gene sets, however, revealed numerous similarities between gene networks. While each treatment was associated with somewhat different significant IPA networks, the merged gene networks for each model revealed similar sets of gene ‘hubs’ (Figures 4, 5, and 6). While these ‘hubs’ were not necessarily altered themselves, they linked dysregulated genes suggesting that there may be some common processes that remain altered into adulthood following developmental ethanol exposure, regardless of timing. Specifically, glutamate receptor subunits appeared as core molecules in all treatment paradigms, as did many neurotrophic molecules. Also, the developmentally-essential gene *Htt* was associated with the altered gene networks of each ethanol treatment model. This is, as far as we are aware, unreported in prenatal alcohol exposure literature; however, it is known that *Htt* has roles in cell survival, proliferation, and migration [101,102]. Further, wild-type *Htt* binds repressor proteins allowing the expression of critical neuronal genes such as *BDNF*[103]. Despite this and its well-established role in neuropathology, its relationship with neurodevelopmental alcohol exposure remains an unexplored avenue, and though its consistent appearance as a hub here is intriguing, an explanation would only be speculative.

### Neurodevelopmental ethanol exposure and long-term gene expression changes

Given that this study examined the long-term effects of neurodevelopmental exposure to ethanol, it may be pertinent to ask what initiates and maintains these gene expression changes over time. A clear answer to these questions is not straightforward and may involve some combination of the primary (acute) and secondary effects of ethanol exposure. Factors that instigate these changes in gene expression may include ethanol-induced apoptosis of susceptible cell types, leading to an overall change in the cellular composition of the brain and, subsequently, the overall pattern of brain gene expression [27,32]. The authors acknowledge that the use of whole brain tissue makes this a possible reason for the results obtained by this study. Also, it is important to note that, while the genes identified may represent a broad response by the neural transcriptome to ethanol, it would be expected that various brain regions respond differently to ethanol exposure given that developmental processes do not occur uniformly and each region would have its own gene expression profile.

Another contributing mechanism may be the ability of ethanol to disrupt developmental processes that are highly reliant on external cues such as cell proliferation, migration, or differentiation [39,58,104]. These mechanisms would represent immediate effects of alcohol that, during adulthood, do not directly alter gene expression *per se* but have altered cellular identity or physiology of the brain such that the appropriate balance of neural gene expression is not maintained. This hypothesis has been suggested for a number of spectrum disorders, and has been referred to as a neurodevelopmental ‘footprint’ of teratogen exposure [15,105,106].

Finally, it is possible that there may be some factors that maintain inappropriate gene expression at the molecular level. Recent reports suggest that developmental ethanol exposure can affect epigenetic patterning, particularly DNA methylation and microRNA expression, which can produce long-term and relatively stable changes to the expression of a number of genes [107-111]. How these changes may occur and how they are maintained is unknown, but this hypothesis is certainly worth further investigating as it would have implications towards the persistence of FASD phenotypes throughout the life of an individual.

## Conclusions

This study was initiated to provide a comparison of the timing effects of neurodevelopmental ethanol exposure on long-term changes in the brain transcriptome. The results show that ethanol causes long-term molecular changes at all developmental stages studied. Also, ethanol treatment, regardless of timing, appears to cause a long-term disruption to genes associated with developmental processes that are active at the time of exposure, resulting in a molecular ‘footprint’ of altered genes that perhaps reflects the most susceptible biological networks at that developmental stage. This disruption likely influences the resulting physiology of the adolescent and adult brain and, subsequently, the phenotypes observed in the exposed individual. Not only does this argue that timing is an important factor in determining the variability and severity of FASD-related traits, but also that knowledge of the developmental stage of exposure may offer a modest predictive value towards the cognitive and behavioral outcomes of an individual prenatally exposed to alcohol. While future experiments are needed to assess the mechanisms underlying the observed gene expression changes, including differences between distinct brain regions and cell types, this model may provide an opportunity to evaluate some of the molecular processes that suffer long-term changes from alcohol exposure at susceptible neurodevelopmental times.

## Competing interests

The authors declare that they have no competing interests.

## Authors’ contributions

MLK and KM performed all mouse work including injections and tissue isolation. RNA extraction was performed by MLK, KM, and RLS. KM performed mouse work, tissue isolation and extractions for trimesters 1 and 2. MLK performed all array and pathway analyses. Gene confirmation was performed by MLK, KM, and RLS. Manuscript was written by MLK with contributions from KM and SMS. Project was developed by MLK, KM, and SMS. All authors read and approved the final manuscript.

## Supplementary Material

Additional file 1: Table S1 Genes identified as differentially expressed in the brain of adult mice following neurodevelopmental alcohol exposure. Lists represent genes identified at P60 following ethanol treatment at E8/11 (trimester 1), E14/16 (trimester 2), and P4/7 (trimester 3). Data included are gene-specific fold-changes and *P*-values. Click here for file
